# Macrocyclic peptides as a new class of targeted protein degraders

**DOI:** 10.1039/d4cb00199k

**Published:** 2025-01-15

**Authors:** Xuefei Jing, Joel P. Mackay, Toby Passioura

**Affiliations:** a School of Life and Environmental Sciences, The University of Sydney Sydney NSW 2006 Australia; b School of Chemistry, The University of Sydney Sydney NSW 2006 Australia toby.passioura@sydney.edu.au; c Insamo South Pty Ltd Chippendale NSW 2008 Australia

## Abstract

Targeted protein degraders, in the form of proteolysis targeting chimaeras (PROTACs) and molecular glues, leverage the ubiquitin–proteasome system to catalytically degrade specific target proteins of interest. Because such molecules can be extremely potent, they have attracted considerable attention as a therapeutic modality in recent years. However, while targeted degraders have great potential, they are likely to face many of the same challenges as more traditional small molecules when it comes to their development as therapeutics. In particular, existing targeted degrader design is largely only applicable to the same set of protein targets as traditional small molecules (*i.e.*, ∼15% of the human proteome). Here, we consider the potential of macrocyclic peptides to overcome this limitation. Such molecules possess several features that make them well-suited for the role, including the ability to induce the formation of ternary protein complexes that can involve relatively flat surfaces and their structural commonality with E3 ligase-recruiting peptide degrons. For these reasons, macrocyclic peptides provide the opportunity both to broaden the number of targets accessible to degrader activity and to broaden the number of E3 ligases that can be harnessed to mediate that activity.

## Abstract 2

Targeted degraders and cyclic peptides are both emerging therapeutic modalities. This review describes recent efforts to combine these approaches, to generate cyclic peptidic targeted degraders with advantages over either parent technology.

## Graphical abstract

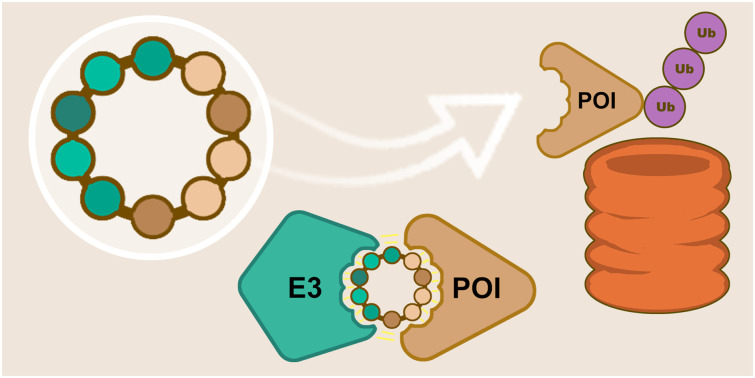

## Introduction

The ubiquitin–proteasome system is the primary mechanism of protein turnover in eukaryotic cells and is involved in a wide range of cellular processes, including cell cycle regulation, inflammation, modulation of signalling pathways, development and differentiation. Mechanistically, it consists of two biochemical steps. In the first step, a target protein to be degraded is covalently linked to multiple molecules of ubiquitin, a highly-conserved 8.5-kDa protein. In the second step, ubiquitinylated proteins are degraded by the proteasome.^1^

The ubiquitylation process is tightly regulated and highly target-specific in order to prevent indiscriminate degradation of cellular components. Hundreds of enzymes have been identified in the ubiquitylation pathway, with the core elements categorised as E1, E2 or E3 proteins.^1^ E1 proteins use ATP to activate ubiquitin, which is then transferred to an E2 protein. An E3 ligase then mediates the transfer of the activated ubiquitin onto the target protein.^2–4^ In humans, there are only two E1 ligases for ubiquitin (a further six activate ubiquitin-like proteins but are not considered here) and ∼40 E2 ligases, but more than 600 E3 ligases.^5–7^

The likely reason that there are many more E3 ligases than E1 or E2 ligases is that the former confer target specificity, and so a wide range of E3 ligases is required to interact with the wide range of proteins that need to be degraded in a specific fashion.^8^ E3 ligases recognize their protein targets through ‘degrons’, peptidic motifs that act as degradation signals.^7,9,10^ Known degrons include native polypeptide sequences, as well as diverse structures resulting from post-translational modifications such as phosphorylation (resulting in the formation of phosphodegrons), hydroxylation, and cyclic imide formation (as observed for the E3 ligase cereblon, *vide infra*).^11–13^ This diversity of degron structures and their cognate E3 ligases allows for finely tuned regulation of protein degradation. For example, the von Hippel-Lindau E3 ligase (VHL, discussed in greater detail below) acts as an oxygen sensor through recognition of a 4-hydroxy-proline containing degron that occurs at normal intracellular oxygen concentrations but not during hypoxia.^14–16^

Over the past decade, it has become apparent that small molecules are capable of both modulating the activity of E3 ligases towards their natural targets and recruiting E3 ligases to target proteins of interest (POIs) beyond their natural range. Such “targeted degraders” can be extraordinarily potent (sub-picomolar activities in some cases^17^), and at least 29 have progressed to clinical trials. Indeed targeted degraders of the IMiD class (discussed in greater detail below) are already in clinical use, though their mechanism of action was not known at the time of approval.^18^ However, existing targeted degrader design paradigms are largely restricted to the same set of protein targets as are traditional small molecules (*i.e.*, ∼15% of the human proteome), and will therefore not be applicable to most disease-related POIs.^19,20^

Macrocyclic molecules, particularly macrocyclic peptides, are capable of targeting a much wider range of POIs than small molecules (including existing targeted degraders).^2,19,21^ As such, they represent a potential pathway to broadening the scope of targeted degraders. In this perspective, we consider the potential of these molecules as a modality for targeted degradation of diverse POIs.

## Molecular glues

The discovery that drug-like molecules could strengthen or induce protein–protein interactions dates back to the early 1990s, when the macrocycle immunosuppressants cyclosporin A, FK506 and rapamycin were shown to induce inhibitory interactions between their respective binding partners and the protein targets.^22–25^

### Cyclosporin A

**Fig. 1:**
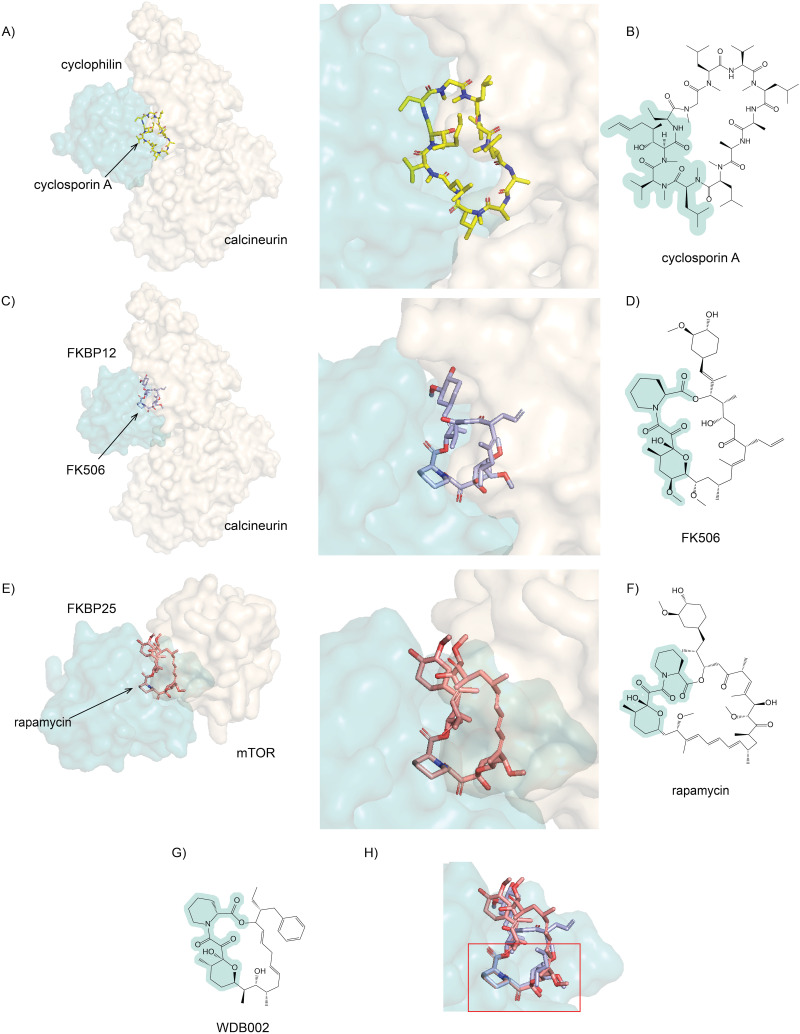
Commonalities and differences in the binding modes of cyclosporin, FK506 and rapamycin. (A) Crystal structure of human calcineurin bound to cyclosporin A (yellow) and human cyclophilin (PDB: 1MF8)^29^ with a zoomed-in interface. (B) Chemical structure of cyclosporin A. (C) Crystal structure of *Aspergillus fumigatus* calcineurin bound to FK506 (light blue) and *A. fumigatus* FKBP12 (PDB: 6TZ7)^30^ with a zoomed-in interface. (D) Chemical structure of FK506. (E) Crystal structure of the FRB domain of human mTOR bound to rapamycin (salmon red) and human FKBP25 (PDB: 5GPG)^31^ with a zoomed-in interface. (F) Chemical structure of rapamycin. (G) Chemical structure of and WDB002. (H) Superimposed structural comparison of FK506 (light blue) and rapamycin (salmon red); a conserved FKBP binding moiety from two macrocycles is indicated in red square.  In each case, the E3-binding moiety is highlighted in pale green.

Cyclosporin A (CsA) is a macrocyclic peptide immunosuppressant that was initially isolated from a soil fungus and is widely used to prevent transplant rejection.^24,26^ Although approved for human use in 1983, CsA's mechanism of action was not fully elucidated until almost a decade later, when it was demonstrated that CsA inhibits the protein phosphatase calcineurin, through the formation of a complex with the peptidylprolyl isomerase cyclophilin^24,27,28^ (Fig. 1(A) and (B)). This mechanism of action (the formation of a complex between two cellular proteins that do not otherwise interact) led to CsA being described as a “molecular glue” – a term that is now generally applied to relatively small molecules capable of inducing the formation of a ternary complex with two biomacromolecules.^24^

### FK506 and rapamycin

Around the same time that CsA was approved for prescription use, FK506 – a natural partially-peptidic macrolide with activity similar to CsA – was discovered in an immunosuppressant screening program using a microbial natural product library. FK506 binds to FK506 binding proteins (FKBPs) and inhibits calcineurin activity by forming a ternary complex with calcineurin (Fig. 1(C) and (D)).^25,32^ Notably, despite having distinct structures and binding partners, FK506 and CsA inhibit the same protein target, calcineurin, demonstrating that different combinations of molecular glue and binding partner can target the same protein of interest. A few years later, the structurally related natural macrolide rapamycin was shown to also bind FKBPs and to induce the formation of a ternary complex with a different target protein, mTOR (the Mechanistic Target Of Rapamycin) (Fig. 1(E) and (F)).^33^ FK506 and rapamycin (which were both extracted from strains of *Streptomyces*) share a conserved FKBP-targeting moiety (Fig. 1(H)), and both are now used as immunosuppressive drugs.^34,35^

It is remarkable that alterations in the non-FKBP binding portion of FK506 and rapamycin allow targeting of completely different POIs through ternary complex formation.^34,35^ An additional example of the ability of FK506 analogues to target diverse POIs is the natural product WDB002, which was also discovered in *Streptomyces*. WDB002 shares the same FKBP recruiting moiety as FK506 and rapamycin but differs from both in its POI-targeting moiety (Fig. 1(G)). This change causes the WDB002-FKBP12 complex to inhibit yet another POI – the human centrosomal protein 250 (CEP250).^36^

Recently, a synthetic approach to the modular development of FKBP-recruiting molecular glues was described.^37^ A focused peptide library was constructed in which all members shared a conserved FKBP-targeting moiety but each member featured a distinct POI-targeting region. This strategy led to the discovery of rapadocin, a nucleoside transporter 1 (hENT1) inhibitor that acts as a molecular glue by forming a ternary rapadocin-FKBP12-hENT1 complex. This study demonstrates that the two binding epitopes of a cyclic peptide molecular glue can be decoupled in a manner that allows modular development of glues targeting new POIs. One potential application of such modularity is in the discovery of macrocyclic targeted degraders, which will be discussed in greater detail later.

### Other natural macrocyclic molecular glues

It has become clear that many natural macrocyclic peptides function as protein–protein interaction (PPI) stabilisers. For example phalloidin,^38^ jasplakinolide,^39^ doliculide,^40^ and chondramide C^41^ are all macrocyclic peptides that bind actin at subunit interfaces and stabilise the actin polymer. As is typical of cyclic peptides, they are more ‘three-dimensional’ than many small molecules and demonstrate high shape complementarity with their respective targets. Because of their size and complexity, they are able to make significant contacts with the (often relatively flat) surfaces of two distinct proteins, providing the potential for high selectivity.^42,43^

All of these macrocyclic peptides were isolated from natural sources. While the endogenous functions of these molecules are not well-understood, it is striking that they are all capable of modulating biological activities through molecular glue mechanisms. This suggests that macrocyclic peptides may be particularly well-suited to the role of stabilisers of PPIs.^43^

### Thalidomide and its analogues

**Fig. 2:**
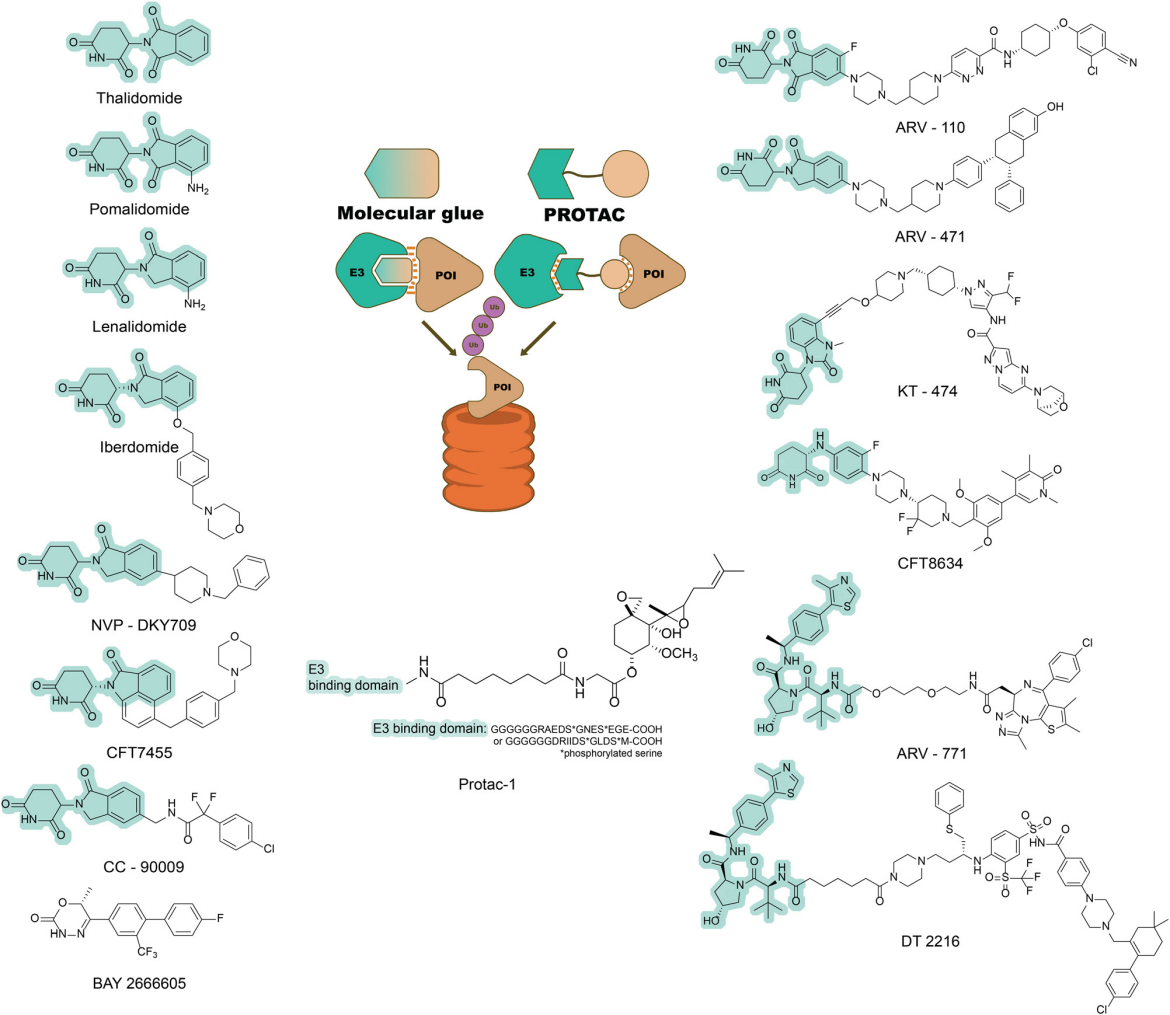
Two forms of targeted degraders (PROTACs and molecular glues). Comparison between molecular glues and PROTACs (centre panel). When bound to an E3 ligase, a molecular glue creates a new interaction surface. This new surface allows the E3 ligase to target new proteins of interest (POIs) and mediate their degradation by the proteasome. PROTACs are bivalent molecules that consist of two ligand warheads. One warhead targets a binding pocket on the POI, while the other warhead targets an E3 ligase, creating a ternary complex that again leads to proteasomal degradation of the POI.^43^ Left-hand side: A selection of known molecular glues approved by the US Food and Drug Administration or undergoing clinical trials by 2022.^50^ Right-hand side: Known chemical structures of PROTACs involved in clinical trials.^53^ In each case, the E3-binding moiety is highlighted in pale green.

Thalidomide was initially prescribed to pregnant women as a sedative in the 1950s but was later discovered to cause catastrophic birth defects. In 1965, interest in thalidomide was revived as it and its analogues (lenalidomide and pomalidomide) were shown to have immunomodulatory and anti-inflammatory activities and were defined as immunomodulatory imide drugs (IMiDs).^44,45^ Little was known about their mechanism of action until 2010, when it was serendipitously discovered that IMiDs primarily bind to cereblon (CRBN) – the substrate-binding subunit of the E3 ubiquitin ligase complex CUL4-RBX1-DDB1-CRBN.^46^ Subsequent work revealed that IMiDs act as molecular glues. Binding of IMiDs alters the surface of cereblon, allowing it to bind and ubiquitinate new, non-native protein substrates (termed neo-substrates) and target them for proteosomal degradation.^47,48^ Many neo-substrates have been identified for the IMiDs, several of which are zinc-finger transcription factors from the Ikaros family.^49^ Since the discovery of the IMiD mechanism of action, many thalidomide derivatives have been developed and several have entered clinical trials or in clinical use (Fig. 2),^50–52^ targeting neo-substrates such as IKZF1 or IKZF3.

The example of thalidomide and its analogues demonstrates that molecular glues can be effective targeted degraders. However, such molecules have proven relatively challenging to develop. This difficulty is primarily due to the lack of a systematic pipeline for molecular glue discovery – which in turn arises from the inherent difficulty of designing molecules to target two proteins simultaneously at what are often relatively flat surfaces. For this reason, the majority of molecular glues have been discovered serendipitously.^51^ This bottleneck currently limits the range of POIs that can be targeted by molecular glues.

## PROTACs

Although the discovery of new molecular glue degraders has proven challenging, another group of molecules has emerged in parallel as targeted degraders that utilize the UPS. Proteolysis-targeting chimeras (PROTACs) are distinguished from molecular glues by the former's clearly heterobifunctional structure, which consists of two (usually small molecule) ligands joined by a linker. One of these ligands targets the POI, while the other recruits an E3 ligase, inducing the formation of a ternary complex, ubiquitination of the POI and subsequent degradation^2,4,54^ (Fig. 2).

The first PROTAC (Protac-1) was made by covalently linking the small molecule ovalicin (a covalent ligand to the methionine aminopeptidase-2 protein [MetAP-2]) and a 19-residue phospho-peptide degron derived from IkappaB kinase α (IκBα), which is recognized by the Skp1-Cullin-F box complex (SCF^βTRCP^) E3 ligase (Fig. 2). Assays in cell lysate showed that ovalicin induces an interaction between MetAP-2 and SCF^βTRCP^ (which would not normally occur), resulting in MetAP-2 ubiquitinylation and subsequent degradation.^55–58^ Protac-1 thus demonstrated the feasibility of systematically designing heterobifunctional molecules to target proteins for degradation *via* the UPS. However, Protac-1's relatively large molecular mass and significant polarity made it incapable of diffusion across cell membranes and hence unsuitable for development as a therapeutic against an intracellular target. A key step in the development of more “drug-like” PROTACs has therefore been the discovery of small-molecule analogues of E3 ligase-recruiting peptide degrons.

The first cell-permeable small-molecule PROTAC was developed by tethering an androgen receptor (AR) ligand (the selective androgen receptor modulator – SARM) to nutlin, an imidazoline based ligand for the E3 ligase MDM2 (mouse double minute 2 homolog). While not highly potent, the resulting SARM-nutlin PROTAC reduced AR protein levels in cultured cells, demonstrating simultaneous targeted degradation and passive cell membrane permeability.^59^

Subsequently, a range of more potent PROTACs that incorporate small-molecule E3 recruiters have been developed. To date, these PROTACs have most commonly utilised either (i) hydroxyproline-containing tripeptides capable of recruiting the von Hippel-Lindau tumour suppressor (VHL) E3 ligase, or (ii) analogues of thalidomide that are capable of recruiting the cereblon E3 ligase as described above^2,3^ (Fig. 2). Variants of E3 recruiters have now been used to make a wide range of PROTACs targeting diverse POIs, and several such molecules are in clinical trials or already in clinical use.^18,53^

Compared to more traditional small-molecule drugs, both PROTACs and molecular glues can be substantially more potent.^17,52,60,61^ This potency appears to derive from their pseudo-catalytic (rather than stoichiometric) mechanism of action, which allows a single drug molecule to promote the degradation of multiple molecules of a POI.^6,62^ Furthermore, compared to molecular glues, PROTACs have proven somewhat more amenable to systematic design. There are now many examples of a small-molecule ligand against a POI being linked to a known E3-recruiting ligand (using linkers of different lengths and rigidities), and the resulting molecule(s) displaying degrader activity.^2,3^

However, while small-molecule PROTACs have demonstrated exciting therapeutic potential, their development faces notable challenges. It has been estimated that only ∼15% of human proteins are targetable using small molecules^19^ and small-molecule PROTACs appear to largely share this limitation.^20^ Existing PROTACs are similar to small-molecule inhibitors in the way that they require a typically well-defined ligand binding site.^2,20^ These two modalities likely share an overlapping set of druggable protein targets, and many of these protein targets already have well-established inhibitors. Thus, there remains great potential for new types of targeted degrader with the capability to target a much wider range of POIs than existing approaches.^20^

## Macrocyclic peptides

Macrocyclic peptides are appealing scaffolds for therapeutic development, with the potential to overcome the limitations of PROTACs (and other small molecules) mentioned above. By virtue of their larger size, they can make strong and selective interactions with relatively large and shallow protein surfaces. This property allows macrocyclic peptides to target a much wider range of POIs, even those without ligand binding pockets. Indeed, the very first molecular glues, such as cyclosporin A, are themselves macrocyclic peptides, demonstrating the capacity of such molecules to bind to POIs for which there are no known small-molecule ligands. Moreover, powerful tools for the discovery of macrocyclic peptides have been developed in recent years, and selection-based methodologies (thoroughly reviewed elsewhere:^63,64^) now enable the rapid identification of macrocyclic peptides with good potency and specificities.^63^ More recently, it has become apparent that macrocyclic peptides identified through display screening approaches can stabilise specific conformations of a POI and even induce protein–protein interactions (discussed below), suggesting their potential as molecular glues and/or targeted degraders.

### MATE transporter stabilization

To the best of our knowledge, the first demonstration that a macrocyclic peptide derived from display screening (rather than a natural product) could stabilise a single conformation of a POI and strengthen protein–protein interactions was the use of the so-called MaL6 peptide as a co-crystallization ligand for the multidrug and toxic molecule extrusion protein from *P. furiosus* (PfMATE). By mixing MaL6 with selenomethionine-derivatized PfMATE, dimerization was induced and crystal quality and reproducibility were greatly improved, allowing the transmembrane protein structure to be determined at 2.5-Å resolution.^65^ This study demonstrated the feasibility of discovering co-crystallising protein stabilisers in the form of macrocyclic peptides through an effective and systematic display system.^66^

### PHD2 oligomerisation

Following on from the success with pfMATE, mRNA display selection was used to identify macrocyclic co-crystallization ligands for the human hypoxia-inducible factor prolyl hydroxylase 2 (PHD2).^67^ Although PHD2 is predominantly monomeric in solution, it preferentially crystallises as a homotrimer. However, in crystal structures reported prior to 2020, the active site of PHD2 was sterically blocked, precluding the study of inhibitors and substrates in complex with PHD2. Chowdhury *et al.* solved this problem by using mRNA display based RaPID selection to identify a macrocyclic peptide, 3C, that promoted PHD2 crystallisation and oligomerization.^67^ In this way, the authors determined structures for both inhibitor and substrate bound forms of PHD2. However, while 3C promoted PHD2 oligomerization in crystalline form, size exclusion chromatography coupled with multiangle laser light scattering (SEC-MALS) revealed that PHD2 remains predominantly monomeric in solution even when 3C is present, indicating that the molecular glue-like mechanism of 3C is restricted to the context of crystal formation.

### Bromodomains

Many early PROTAC studies involved bromodomain and extra-terminal domain (BET) family proteins. The BET family comprises four proteins (BRD2, BRD3, BRD4 and BRDT) that regulate transcription through recognition of acetylated lysine residues *via* a tandem pair of bromodomains (BDs; BD1 and BD2).^68,69^ These proteins have since become a model system for the development of novel targeted degraders, owing to a combination of the clinical potential of their inhibition and the availability of small molecules that bind these bromodomains. JQ1 is the archetypal BET-protein inhibitor and, despite its lack of selectivity across BET-family members, it has been widely used as a BET-targeting warhead in many PROTAC studies.^70,71^ For example, MZ1 – one of the first PROTACs made entirely of non-peptidic small molecules – tethers JQ1 to a VHL E3 ligase recruiting ligand. MZ1 successfully mediated degradation of BET proteins, with a (perhaps unexpected) preference for BRD4 over BRD2 and BRD3.^72^ A cyclized version of MZ1, created using structure-based design, displayed similar efficacy and even enhanced selectivity against BD2s of BRD2 and BRD4.^73^

A more recent study used mRNA display-based RaPID screening to identify macrocyclic BRD4 ligands closed by a bipyridyl moiety that exhibited limited cell permeability.^74^ By linking these molecules to proteosome targeting Arg–Arg–Arg–Gly motifs, the authors were able to generate PROTAC-like molecules with some capacity to induce degradation of BRD4 in cultured cells, albeit with fairly low potency. Nonetheless, this study is significant because it represents the first use of a display selection-derived macrocycle as the targeting moiety of a PROTAC-like bipartite targeted degrader.

**Fig. 3:**
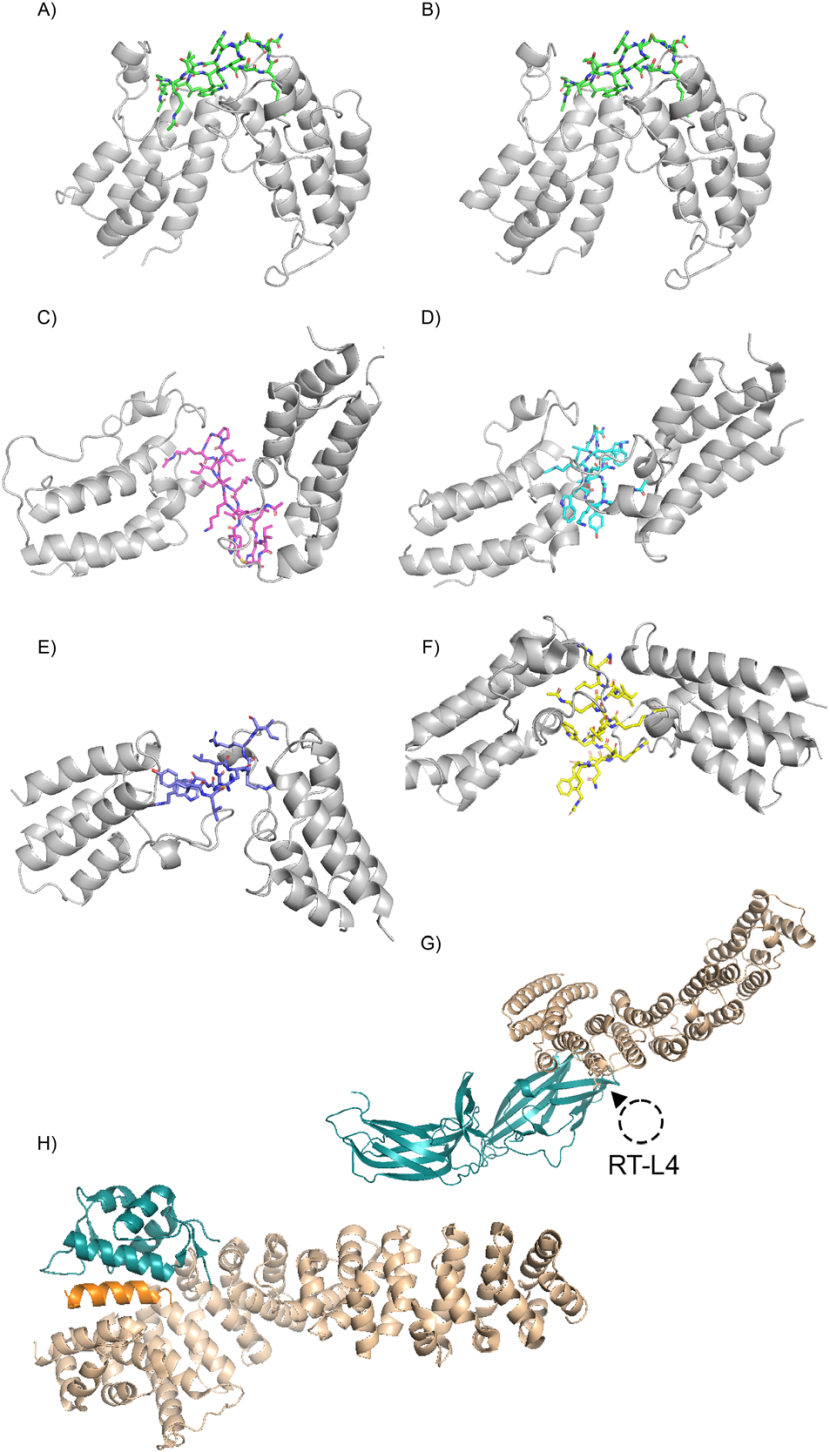
Crystal structures of protein complex-inducing macrocyclic peptides. (A) 3.1B (green) + BRD4-BD1 (PDB: 6U74); (B) 3.1B (green) + BRD4-BD2 (PDB: 6U8G); (C) 3.2C (magenta) + BRD3-BD2 (PDB: 6ULP); (D) 3.2A (cyan) + BRD4-BD1 (PDB: 6U8M); (E) 4.2C (purple blue) + BRD2-BD2 (PDB: 6ULT); (F) 4.2A (yellow) + BRD4-BD1 (PDB: 6ULV).^75^ (G) Vps26 and Vps35 complex structure (PDB: 6VAC) with binding site for RT-L4 (indicated by a dashed circle) based on cryoEM data (EMDB: 24963).^77,78^ (H) Crystal structure of the stapled helix Helicon H330 (orange) in complex with MDM2 (residue 17–111) and β-catenin (residues 134–665) (PDB: 8EIC).^79^

Similar to the examples of PfMATE and PHD2 described above, our own (T. P. and J. M.) work has demonstrated the capacity of macrocyclic peptides to induce homomeric interactions. As part of efforts to generate macrocyclic ligands to the BET BDs using mRNA display based RaPID screening, we identified multiple macrocyclic peptides that appeared to induce homomeric interactions between these domains.^75,76^ In these studies, acetyllysine (KAc) was included in the invariant scaffold or in the randomized region, in an effort to target the canonical KAc binding pocket of the domains. Crystal structures of several BD–peptide complexes revealed ternary complexes involving two BD bound to each cyclic peptide (Fig. 3(A)–(F)). Moreover, SEC-MALS analysis revealed that one cyclic peptide (3.1B) was able to induce dimerization of the BD1 domain from BRD4 in solution, as well as in the crystal structure.

### Stabilisation of the retromer complex

The examples above demonstrate the capacity of macrocyclic peptides to stabilise homomeric interactions. However, applications of molecular glues and PROTACs typically require the stabilisation/induction of heteromeric interactions. By performing mRNA-display based RaPID selection against a stable trimeric complex (the Vps35–Vps26–Vps29 complex, itself a subset of the human retromer complex), Chen *et al.* identified a macrocyclic peptide (RT-L4) that acts as a molecular glue to stabilise the trimeric target complex.^78^ Biophysical and mutagenesis data showed that RT-L4 specifically bound to the interface between the Vps26 and Vps35 subunits, a finding further supported by low-resolution cryo-electron microscopy data (Fig. 3(G)). Moreover, RT-L4 was shown to increase the binding affinity of the Vps35–Vps26–Vps29 complex for several known interacting proteins, suggesting that its molecular glue activity could modulate biological processes and that its mechanism of action is therefore comparable to natural product macrocycles such as cyclosporin and rapamycin. As such, RT-L4 likely represents the first laboratory derived peptide macrocycle that can act as a molecular glue to stabilize a heteromeric protein interaction.

### Stapled helices

Very recently, stapled α-helices (a sub-class of macrocyclic peptides) capable of acting as molecular glue targeted degraders have been described.^79^ These molecules were identified through a tandem phage display approach, in which initial screens were conducted against nine different types of E3 ligases and the discovered E3-ligase ligands were then used to guide the generation of focused libraries (in which residues essential for E3-binding were conserved) for phage display screening against proteins of interest.^79,80^ This two-step screening process yielded multiple molecular-glue-like molecules, several of which were shown to induce ternary complex formation between E3 ligases and POIs (Fig. 3(H)). While this study did not demonstrate degradation of target proteins inside cells (presumably because the stapled helices described were not membrane permeable), it demonstrated for the first time the utility of macrocyclic peptide display screening to identify both novel E3 ligase ligands (through display screening of unbiased libraries) and molecular glues (through screening of biased cyclic peptide libraries).

## The potential of macrocyclic peptides as protein degraders

The studies described above demonstrate that macrocyclic peptides can bind flat surfaces with high affinity and specificity, and also induce the formation of ternary complexes. These two characteristics hint at their potential as next generation targeted degraders for POIs that are inaccessible to current degrader design approaches. Other lines of evidence also suggest macrocyclic peptides may be particularly useful in this context.

### Degrons are peptidic

Natural peptides have played a critical role in the development of targeted degraders. The first PROTAC (Protac-1) was derived from a degron peptide, and in fact all PROTACs are peptidomimetic to some degree, since they mimic the (peptidic) degrons that are the natural ligands for E3 proteins. This point is clearly demonstrated by the examples of the two mostly commonly used small-molecule E3 recruiters (and their analogues), which target the VHL and cereblon E3 ligases.

**Fig. 4:**
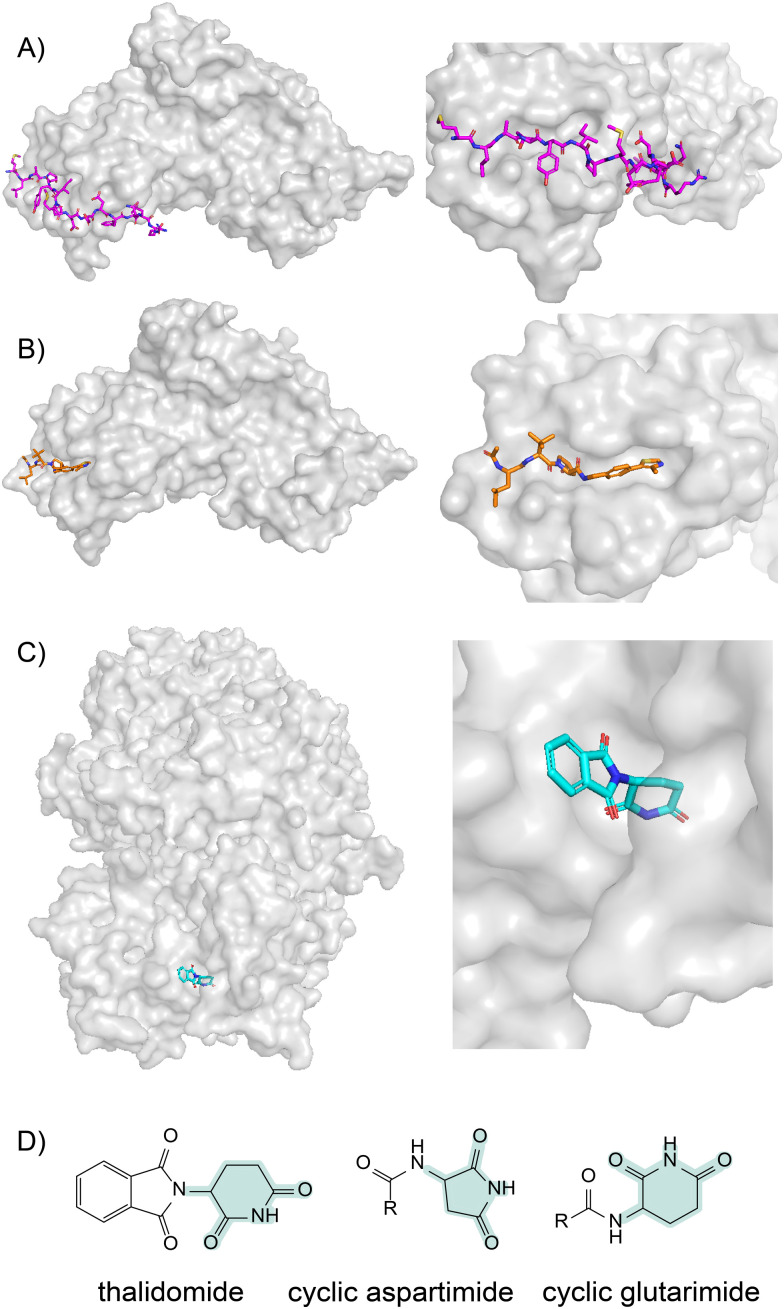
E3 ligase recruiting peptide degron and small-molecule degron mimic (A) Structure of a hydroxylated HIF-1a peptide (magentas) bound to the pVHL–ElonginB–ElonginC complex (PDB: 1LM8).^15^ (B) Structure of the pVHL–ElonginB–ElonginC complex bound to a HIF-1a small-molecule mimic coloured in orange (PDB: 4W9J).^82^ (C) Structure of the DDB1-CRBN complex bound to thalidomide coloured in cyan (PDB: 4CI1).^83^ (D) Comparison of the structure of thalidomide and the cyclic imides that constitute the natural degron of cereblon (CRBN). The functional imide moieties that recruit cereblon for UPS initiation are highlighted in green.

The degron peptide for VHL was discovered in 2002, when it was shown that the hypoxia-inducible factor-1 (HIF-1) protein subunit contains a post-translationally hydroxylated proline residue that is required for binding by VHL (Fig. 4(A)).^14,15^ Through medicinal chemistry studies, this hydroxyproline-containing peptide was subsequently transformed into a small molecule with nanomolar affinity for VHL (Fig. 4(B))^81,82^ and has now been used in PROTACs targeting a wide range of different POIs. Notably, despite extensive medicinal chemistry studies, this small molecule is still essentially a tripeptide, demonstrating that its peptidic nature is required for VHL recruitment.

Although cereblon recruiting small molecules (thalidomide and analogues) have been known for many years, the natural degron for cereblon has only recently been discovered. Two 2022 reports demonstrated that the natural substrates for cereblon are C-terminal imides that arise from cyclisation of glutamine or asparagine residues.^12,13^ Viewed in this context, the peptidomimetic character of thalidomide analogues becomes clear (Fig. 4(C) and (D)).

Currently, the VHL and cereblon ligands are the most widely used E3 recruiters in the targeted degrader field. There are, however, over 600 E3 ligases known to function in human cells, which represent a large untapped resource for the design of new and tissue-specific protein degraders. With growing recognition of the need for additional E3 ligases to expand the scope of targeted degradation, identifying and utilising new E3 degrons has become an emerging field of study.

### New macrocyclic targeted protein degraders

The peptidic nature of natural degrons suggests a significant opportunity for targeted degraders based on macrocyclic peptides. To date, peptide degrons have been identified for at least 20 E3 ligases.^16^ In addition, identification of novel macrocyclic peptide ligands to E3 ligase enzymes has been proven to be relatively straightforward using display technologies.^79,84^ For these reasons, we anticipate that such approaches will be used to develop new classes of macrocyclic peptide targeted degraders. We envisage this occurring through two related but distinct approaches.

**Fig. 5:**
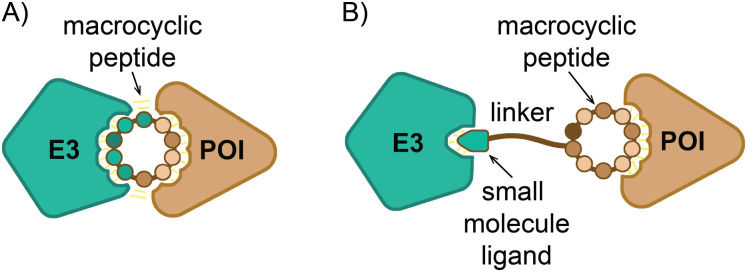
Two types of macrocycle targeted degraders. (A) A macrocyclic peptide that acts as a molecular glue to induce ternary complex formation with a POI and an E3 ligase. (B) A POI-targeting macrocyclic peptide joined by a linker to an E3 ligase recruiting degron (or small-molecule ligand), forming a macrocycle-E3 ligand PROTAC.

First, molecular glues mechanistically comparable to FK506, rapamycin and cyclosporin, but which engage E3 ligases to direct target degradation could be developed (Fig. 5(A)). Thus, cyclic-peptide libraries could be constructed to include a known peptidic degron in combination with randomized elements and panned for affinity to a POI. Indeed, the example of stapled helices identified by Tokareva and colleagues described above is representative of this type of strategy.^79^ Because many degrons are already known,^10,16,85^ parallel screens using separate libraries that each feature a distinct degron would be feasible. Alternatively, screening approaches are conceivable that would directly identify macrocyclic molecules capable of inducing ternary complex formation. Such approaches would, in principle, yield molecular glue-like molecules (although distinct from existing molecular glues, in that they would contain known E3 ligase domains and would therefore be somewhat analogous to PROTACs as well) that can induce degradation of a target POI *via* a chosen E3 ligase (even one with no known degron) in a single step. These related approaches would have the advantage that ternary complex formation would be selected for as part of the screening process. This should obviate the need for extensive optimisation of the linker between the E3 binding and POI binding moieties, as is currently required for most PROTAC design.

Secondly, macrocyclic peptides could also be used simply as the POI-targeting moieties for heterobifunctional PROTAC-like molecules. In such applications, macrocyclic peptides selected (most likely in a display screen) to bind a POI would be fused to degrons or other degradation inducing ligands, generating macrocycle-E3 ligand PROTACs with the capacity to target otherwise undruggable POIs for degradation (Fig. 5(B)). The BET degraders recently described by Chen *et al.*^74^ are the first demonstration of this approach. The design of such macrocycle-E3 ligands promises to be relatively straightforward, given that the discovery of macrocyclic ligands to POIs by display screening and the design of PROTACs through addition of E3 ligands are now both well-established techniques.

It is worth noting that the molecules envisaged above would be compatible with existing medicinal chemistry approaches for the optimisation of peptidic drugs.^86–88^ They would, therefore, fit well into current pharmaceutical pipelines facilitating clinical development. Such optimisation could include fine-tuning the binding affinity of the macrocycle for the E3 ligase and POI separately, since while high affinity for the E3 ligase is desirable, high affinity for the POI could lead to degradation of the macrocycle itself along with the POI.

### The challenge of delivery

The major obstacle to the development of *de novo* macrocycles of any type is that they often exhibit poor oral availability and cell membrane permeability.^64^ Many strategies have been trialed to resolve these pharmacokinetic issues, and they fall into three general categories: (i) diffusion mediated by amphipathic molecules (*e.g.*, cell penetrating peptides); (ii) carrier-mediated or active transport across membranes; and (iii) passive diffusion across membranes (in the manner of small molecules).

So-called cell penetrating peptides (CPPs) are typically amphipathic molecules composed of 5–30 amino acids with a high frequency of arginine and/or lysine residues. CPPs appear to use a variety of internalisation mechanisms, including direct membrane penetration and endosomal uptake *via* endocytic pathways.^89–91^ Many such peptides have been identified, and in some cases, they are capable of delivering biologically active cargos into cells. While potentially applicable as vectors for clinical use, the efficacy of CPPs is highly cargo dependent. Moreover, they often act through mechanisms that require micromolar extracellular concentrations before penetration is observed.^92,93^

Carrier-mediated delivery of otherwise non-membrane permeable agents (using, for example, viral, lipid or nanoparticle delivery systems) are in principle capable of resolving the issue of cellular permeability.^94–98^ However, to the best of our knowledge, all such approaches lead to limited and heterogenous delivery of cargos *in vivo*. Viral gene therapies, for example, are only applicable to diseases where the insertion of therapeutic payload into a small number of cells (rather than all or most of the cells of a patient) can alter the disease progress. Clinical delivery of nucleic acid cargos such as interfering RNA or mRNA vaccines through the use of lipid nanoparticles similarly appears to rely on delivery to a limited number of cells. However, given the current intense interest in this area, it is certainly possible that significant advances in delivery efficiency and cellular targeting will emerge in the foreseeable future.^99^

Finally, intrinsic passive membrane permeability is the mechanism by which small-molecule drugs (including macrocycles such as cyclosporine, FK506, *etc.*) typically achieve oral availability and cell penetration (though such molecules are also often substrates for proteins such as transporters or efflux pumps), and the physicochemical characteristics that underlie passive membrane permeability are well-established (Lipinski's rule of 5). Such rules, however, are difficult to apply to macrocyclic molecules, which can adopt ordered conformations that alter their physicochemical properties.

Despite these challenges, there have been some notable recent successes in this field. For example, an orally available macrocyclic peptide inhibitor of proprotein convertase subtilisin/kexin type 9 (PCSK9), MK-0616, is about to enter phase III clinical trials for the treatment of hypercholesterolaemia. MK-0616 was derived from an initial display screening hit and made orally available through extensive chemical modifications and formulation.^100^ Similarly, LUNA18 is an mRNA display-derived 11-residue macrocyclic peptide inhibitor of the intracellular oncology target K-RAS, which is in early stage clinical trials.^101^ Furthermore, recent computational modelling approaches have demonstrated that relatively large (up to 12mer) membrane permeable cyclic peptides can be predicted *de novo*.^102^ Taken together, these reports suggest that the era of membrane permeable cyclic peptide drugs may finally be dawning.

It is worth noting that, like most macrocycles, PROTACs also tend to violate Lipinski's rule of 5, and it is possible that PROTACs with macrocyclic POI targeting moieties (which have higher molecular masses than either macrocycles or PROTACs) may be very difficult to deliver intracellularly. However, the catalytically mechanism of action of targeted degraders may help to overcome the relatively poor membrane permeability of such large macrocycle PROTACs. The outstanding potency observed for several targeted degraders (described in more detail elsewhere^17,52,60,61^) potentially means that very few molecules need to be delivered intracellularly to have an effect, and so even marginal membrane permeability may be sufficient in this scenario for clinical utility.

## Conclusion

With multiple PROTACs and molecular glues entering clinical trials, there is no doubt that targeted protein degradation is likely to emerge as a *bona fide* therapeutic modality in the coming years. However, the first generation of PROTACs are relatively small molecules and exhibit the same limitations as other small molecule drugs; most notably, they are only applicable to a limited set of targets. Macrocyclic peptides have the potential to turbo-charge the discovery of targeted degraders. The ability of cyclic peptide display systems to yield potent and selective ligands for a POI – even one that lacks a clear binding pocket – represents an opportunity to broaden the reach of PROTACs. Perhaps more excitingly, the high degree of complementarity between these display systems and natural peptide degrons hints at the single-step discovery of cyclic-peptide based molecular glues that act as potent and selective degraders. Although the intracellular delivery of such molecules represents a challenge, the development of approaches to deliver peptides is a highly active area of research. Overall, we believe that macrocyclic peptides are well-suited as a scaffold for the development of targeted degraders against otherwise undruggable targets and could come to the fore as part of a second generation of PROTACs with wider therapeutic utility.

## Data availability

No primary research results, software or code have been included and no new data were generated or analysed as part of this review.

## Conflicts of interest

TP is an employee of and shareholder in, Insamo, a biotechnology company developing macrocyclic peptides for pharmaceutical applications.
